# Comparative evaluation of lung ultrasound versus chest X-ray for pneumothorax assessment post-invasive intrathoracic procedures: A case-costing evaluation

**DOI:** 10.1097/MD.0000000000041959

**Published:** 2025-04-25

**Authors:** Carter Winberg, Ross Prager, Chong Sung Kim, Matthew Meyer, Robert Arntfield

**Affiliations:** a Division of Critical Care, Western University, London, ON, Canada; b Decision Support Analyst, London Health Sciences Centre, London, ON, Canada; c Department of Epidemiology and Biostatistics, Western University, London, ON, Canada.

**Keywords:** chest X-ray, cost, economics, lung ultrasound, pneumothorax

## Abstract

**Background:**

Persistently increasing healthcare spending, paired with growing healthcare demand, highlights the need to identify mechanisms for cost savings. Chest radiography (CXR) is commonly performed following intrathoracic procedures to rule out pneumothorax (PTX) even if the clinical pretest probability is low. However, lung ultrasound (LUS) is known to have superior sensitivity, possibly representing a promising cost-saving tool. In response, we conducted an economic analysis comparing LUS and CXR to exclude PTX after invasive intrathoracic procedures.

**Methods:**

A retrospective review of the radiology case-costing center was performed at an academic cardiothoracic surgical institution to identify the activity and cost of CXRs performed to rule out PTX following intrathoracic procedures. This cost was then compared to the theoretical cost of LUS.

**Results:**

CXRs performed to rule out iatrogenic PTX were common with 22,274 radiographs completed and were economically burdensome, with an associated cost of $1.4 million. Portable CXR cost $75.46 per test, while CXR posteroanterior/lateral costs $41.64. Comparatively, LUS cost $38.38. Implementation would lead to cost savings of $559,537.10 or $41.58, on average, per patient.

**Conclusion:**

Given the superiority of LUS in terms of sensitivity and accuracy for PTX diagnosis, these findings underscore the compelling rationale for its broader integration into clinical practice.

## 1. Introduction

Pneumothorax (PTX) arises from the abnormal accumulation of air between the visceral and parietal pleura. While some PTXs occur spontaneously, the majority are iatrogenic secondary to invasive procedures or surgical interventions.^[1]^ Despite a relatively low incidence of approximately 5 per 10,000 hospital admissions, failure to diagnose PTX can lead to death.^[1]^ Clinicians frequently use imaging to rule out PTX following invasive thoracic procedures, even in patients with a very low pretest probability. Consequently, this practice requires considerable healthcare resources, often with limited diagnostic yield.

Computed tomography (CT) is the gold standard for the diagnosis of PTXs, but chest X-ray (CXR) is more commonly used due to its portability, reduced radiation exposure, and perceived lower cost.^[2]^ However, this diagnostic pathway has been disrupted by recent meta-analyses showing lung ultrasound (LUS) to exhibit superior diagnostic accuracy for PTX (sensitivity 85.7%, specificity 95.3%) compared to CXR (sensitivity 71.4%, specificity 100%).^[3–5]^ This finding is consistent across multiple studies including operators with diverse backgrounds and expertise.^[6]^

LUS has several additional benefits compared to CXR, such as avoidance of ionizing radiation, portability for bedside use, and capability for real-time intervention.^[7]^ Despite these benefits, the widespread adoption of LUS has been limited, in part due to concerns regarding costs and resource allocation at the institutional level. Understanding the cost of adopting LUS compared to CXRs would help decision makers evaluate its potential integration into diagnostic protocols.

Persistently rising healthcare spending, paired with growing healthcare demand, emphasizes the need to identify mechanisms for cost savings.^[8]^ With its known superiority as a rule-out test, LUS could represent a promising cost-saving tool for institutions to adopt. In response, we conducted an economic analysis comparing LUS and CXR to exclude PTX after invasive intrathoracic procedures at an academic cardiothoracic surgical institution.

## 2. Materials and methods

### 2.1. Study design and setting

From the perspective of a single-payer healthcare system funded by federal transfers through the Ontario Health Insurance Plan (OHIP), we retrospectively evaluated the activity and economic burden of CXR use to rule out PTX following invasive thoracic procedures. We then compared total CXR costs to the theoretical cost of adopting an LUS diagnostic pathway, as these cost data were not retrospectively available at our center. This review was conducted at a single institution comprised of 2 tertiary academic hospitals in Ontario, Canada. This study was approved by the Western Research Ethics Board at both sites (Approval #: 116838).

### 2.2. Patient population and data collection

We collected data from adult (age > 18 years) patients who underwent CXR following invasive intrathoracic procedures from January 1, 2020 to December 31, 2022. We included patients from all departments of the hospital (emergency, inpatient, clinic, and day surgery). This population of patients was chosen because of the high burden of CXRs, many undergoing daily CXR during admission, or requiring serial CXR during different stages of chest tube management due to standard practice rule out PTX chest imaging. Additionally, we were able to identify this patient sample more accurately through procedural codes associated with thoracentesis, chest tube insertion, and various cardiothoracic surgeries (Table S1, Supplemental Digital Content, https://links.lww.com/MD/O721). To ensure comprehensive inclusion of the study population, we also conducted a 12-term search of manually entered indications for CXR orders (Table S2, Supplemental Digital Content, https://links.lww.com/MD/O722).

At our institution, Decision Support prospectively stores patient and clinical information from Cerner Electronic Medical Records for all patient encounters. From the Decision Support data warehouse, the absolute total number of CXRs performed, as well as demographics (age and sex), medical imaging details (type of X-ray performed, location performed), and clinical data (intervention before CXR and length of stay) from our sample population were provided. All retrospective research data utilized in this study were encrypted and de-identified with strict adherence to established research standards. Duplicate entries are removed from the dataset.

Alternatively, point-of-care ultrasound (POCUS) costs are not captured in the decision-support warehouse. The theoretical cost for LUS was compiled from the incurred costs of the critical care department that independently implemented LUS in the clinical workflow. Current POCUS scans were extracted from the quality assurance software used. The costs were matched to the CXR costs, as outlined below.

### 2.3. CXR cost allocation

The diagnostic imaging costs were provided from the Decision Support service at the institution. Radiographic costs are identified by the institution through time-driven activity-based costing where variable and fixed costs associated with the completion of each diagnostic test are combined with a time-based multiplier and summated to identify total cost to the institution. Institutional costs are routinely cross-referenced with internal financial records to ensure accuracy. Institutional indirect costs, both variable and direct, associated with CXR were excluded because of challenges aligning them to LUS costs. The OHIP billing code fee is included to capture the complete cost of the system. Chest anteroposterior portable and chest anterior/lateral portable were combined into a single category labeled CXR Portable. Chest posteroanterior/lateral (PA/Lat) was categorized separately owing to their significantly different costs. Due to minor pricing variations across the fiscal years under review, the costs were averaged.

### 2.4. LUS cost allocation

As mentioned, the cost of LUS is not managed through the institution’s cost center; therefore, we developed a theoretical model based on anticipated costs within analogous domains to CXR. We structured the costs of LUS to model how an institution can adopt POCUS to effectively replace the number of identified CXRs, including sufficient hardware (ultrasound devices), software (online quality assurance workflow manager), disposables, and operator time. For appropriate coverage of the intensive care unit, emergency department, thoracic surgery ward, cardiac surgery ward, internal medicine ward, and operating rooms/post anesthetic care unit we included cost of purchasing 6 ultrasound machines amortized over an expected lifespan of 5 years. Additional costs included disposable items (ultrasound gel and cleaning wipes), subscription to workflow management software for quality assurance, and OHIP billing code. For the best cost-matching, the same activity-based cost multipliers applied to CXR were applied to LUS costs with an estimated 15 minutes of time for completion of LUS.^[9,10]^

### 2.5. Analytics and assumptions

Given the complexity of cardiothoracic surgeries and patient pathology, which sometimes require multiple CXRs that might not be amenable to replacement with LUS (e.g., assessing for chest tube position), we restricted the number of CXRs considered in our analysis to a maximum of 2 per patient encounter. Although this introduces uncertainty, this was felt to be a reasonable assumption as LUS would be a validated tool to at the minimum replace the postinsertion and postremoval CXRs that are focused on PTX rule out. All costs in the evaluation are reported in Canadian Dollars, with average conversion rates from January 1, 2021, to January 1, 2022.

## 3. Results

### 3.1. Patient demographics

Between January 1, 2020, and December 31, 2022, a total of 11,480 patients representing 13,457 unique visits were included in our analysis. Patients’ mean age was 59.6 years old (SD 20.98), 4248 (37%) were female, and mean hospital length of stay was 9.5 days (SD 17.0). 9260 (68.8%) were inpatient visits (Table 1).

**Table 1:** Demographics and clinical data of study sample population.

Number of patients	Number of visits	Mean patient age, yr (SD)	Sex (% female)	Mean hospital LOS, d (SD)	Inclusion criteria*				Visit method†			
					Surgery	Chest tube	Thoracentesis	Keyword‡	Clinic	Emergency Dept.	Day surgery	Inpatient
11,480	13,457	59.6 (20.98)	37%	9.5 (17.00)	5669	2875	1520	7853	1743	2437	17	9260

LOS = length of stay.

* This can sum to more than total number of visits because a single visit may include more than 1 type but is limited to a single data row during analysis.

† If a patient presented to the ED but was admitted, this record is labeled an “Inpatient” visit. For ED visits without admission, these are labeled “ED”.

‡ Keywords include “CT removal,” “off suction,” “on suction,” “heimlich,” “CT insitu,” “CTx2,” “CT reposition,” “CT daily,” “chest tune,” “CT bilaterally,” “PTX,” “PNX”.

### 3.2. Activity of CXR to rule out PTX

**Figure 1.**
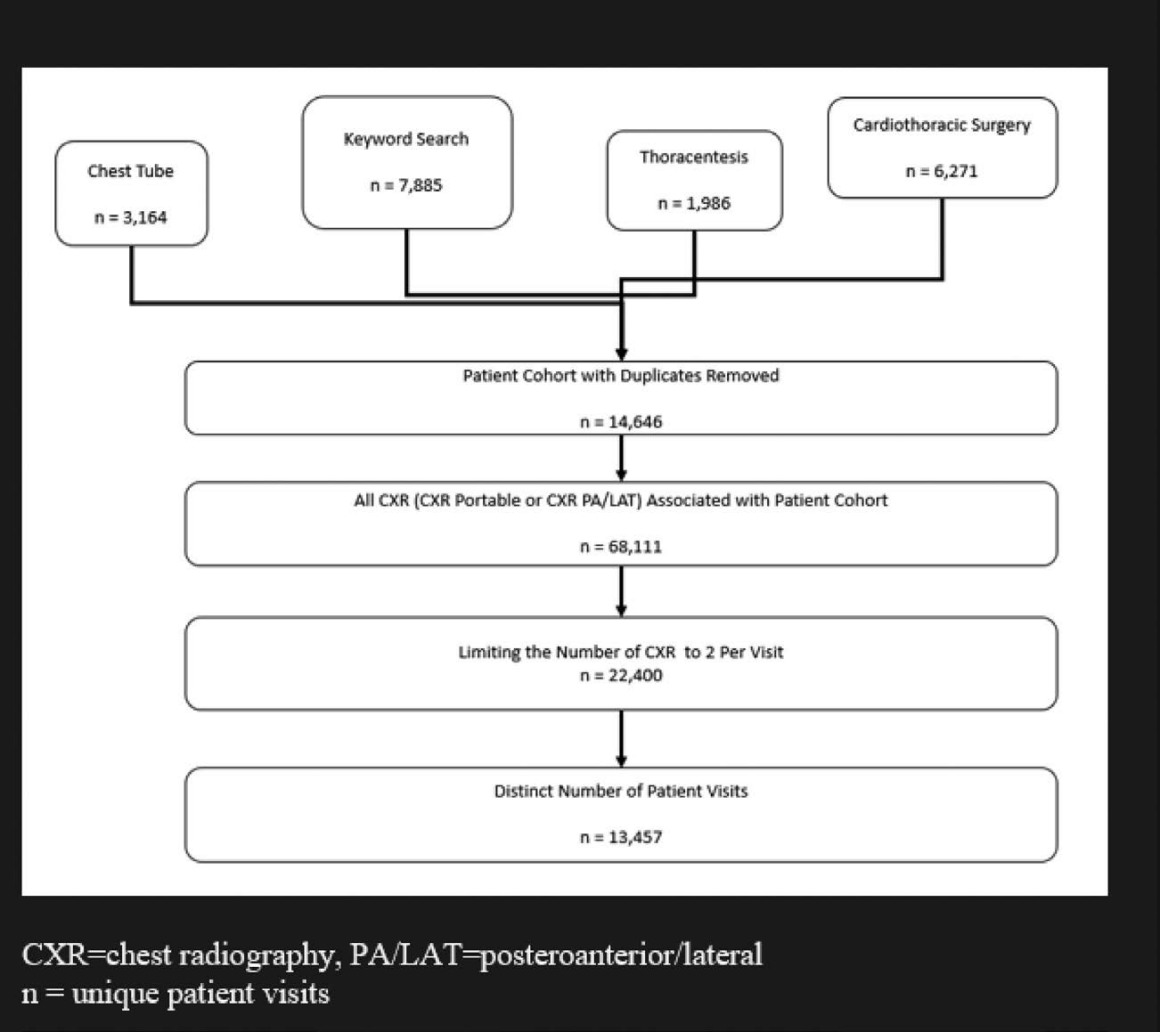
Patient identification and inclusion flow chart.

Our initial search captured 2875 patient encounters identified to have a CXR completed post drainage of the pleural cavity with a chest tube, 1520 following thoracentesis, 5669 after cardiothoracic surgery, and 7853 patients were identified based on the keyword search. After duplicates were removed, a total of 13,457 patients were included. This corresponded to a total of 68,111 CXR completed during these encounters, which was reduced to 22,273 based on our assumption that only 2 CXR completed per encounter were focused on ruling out PTX or chest tube removal (Fig. 1). Most of these studies were portable (13,066) compared to 9207 CXR PA/LAT (Table 3). A total of 264,546 CXR were completed for all clinical indications at the institution;, therefore CXR to rule out iatrogenic PTX represented 8.4% of all CXR conducted during this time.

### 3.3. Cost of CXR

The cost of a CXR Portable was $75.46 and the cost of a CXR PA/Lat was $41.64 (Table 2). These costs incorporate (1) direct labor, including physician cost to the system represented by OHIP billing code and personnel salaries, (2) direct material for necessary supplies, and (3) fixed direct costs, including equipment and software costs. A major contributor was variable direct labor, presumably from radiation technologist’s salary and benefits, representing $47.07 (62.4%) for CXR Portable and $21.13 (50.7%) for CXR PA/Lat. Additionally, the CXR Portable carried higher fixed direct costs from equipment expenses and upkeep. During our study period, a total cost of 1.4 million dollars was attributed to CXRs performed to rule out iatrogenic PTX (Table 3).

**Table 2:** Cost breakdown of CXR compared to theoretical costs of LUS.

Contributing costs	Components attributed to CXR*	Cost contributing to CXR		Components attributed to LUS	Cost contributing to LUS§
		Portable CXR†	PA/LAT CXR‡		
Direct labor					
Physician labor cost	Provincial Government Radiologist Billing Code∥: X090 (single view) X091 (2 views)	$6.35	$10.70	Provincial Government Physician LUS Billing Code: J125	$24.55
Variable direct labor	Unit-producing personnel salary and benefits Medical purchased service Contracted-out expenses	$47.07	$21.13	Software: subscription to workflow manager¶	$2.40
Direct material					
Variable direct material	All supplies directly attributed to a patient (surgical implants, drugs, food, etc)	$1.96	$0.90	Disposables including ultrasound gel, cleaning wipes	$2.00
Fixed direct#					
Fixed direct	Labor: Management & support salary & benefits, salaried physicians Building: Equipment expenses Undistributed building and grounds that is, equipment leases/rental, renovation. Other: Sundry that is, postage, travel, software, catering	$20.08	$8.91	Hardware costs of ultrasound machines**^,^††	$9.43
Total CXR cost:		$75.46	$41.64	Total LUS cost:	$38.38

CXR = chest X-ray, LUS = lung ultrasound, PA/LAT = posteroanterior and lateral.

* Due to case-costing methods these are the most-detailed cost attributions available for diagnostic imaging.

† Costs associated with CXR portable exams are averaged case-costing values for 88,844 exams observed during the study period with an average exam time of 37.9 min.

‡ Costs associated with CXR PA/LAT are averaged case costing values for 102,381 exams observed during the study period with an average exam time of 17.1 min.

§ Estimated costs associated with LUS for an average exam time of 15 min.

∥ Billing codes all represent technical fees cited from Ontario Government schedule of fees.

¶ Licensing cost for subscription to workflow manager that allows for ultrasound training and quality assurance. Currently at our center this cost is $10 000 for coverage of 7 departments.

# CXR fixed direct costs include a time-based multiplier of $0.52/min. In order to best match costs, it was assumed that LUS may include some of same fixed direct costs and therefore the time-based multiplier was added to the LUS hardware costs as well.

** Assuming the need to purchase 6 ultrasound machines to cover the necessary wards. Assuming an approximate cost of $30,000 amortized over expected 5-yr lifespan.

†† 15 min used as exam time for time-based multiplier.

**Table 3:** Cost and activity of CXR performed to rule out PTX following invasive thoracic procedures.

CXR type	Cost per exam ($)	Inclusion criteria*				CXR completed	Total cost ($)
		Thoracentesis	Chest tube	Surgery	Keyword		
Portable	$75.46	209	2747	8595	1515	13,066	$985,960.36
PA/Lat	$41.64	893	1504	2432	4378	9207	$429,414.48
						22,273	$1415,374.84

CXR = chest X-ray, PA/Lat = posteroanterior/lateral, PTX = pneumothorax.

* As some patients fulfilled multiple inclusion criteria a hierarchy of Surgery > Chest Tube > Thoracentesis > Keyword was used for populating the table.

### 3.4. Cost of LUS

The cost of LUS per scan was determined to be $38.38 (Table 2). Based on market research, each ultrasound machine was estimated to cost $30,000 and have a lifespan of 5 years, resulting in an annual cost of $6000 per machine.^[11]^ The total annual cost for 6 machines was calculated at $36,000 and then divided by the total number of CXR performed to rule out PTX to determine the hardware cost per LUS. The subscription to an ultrasound image workflow manager that facilitates clip upload, storage, and quality review is priced at $10,000 annually.^[12]^ In 2020, a total of 4205 ultrasound scans were uploaded to this workflow manager leading to a cost of $2.38 per scan. An estimated $2.00 per scan was attributed to the disposable products like ultrasound gel and cleaning wipes. Lastly, the OHIP billing code for thoracic ultrasound of $24.55 was included to compensate for physician time.

### 3.5. Cost comparison of LUS and CXR

The majority of CXRs ordered were portable, which cost $75.46 per test, while CXR PA/LAT were found to cost $41.64 (Table 3). The implementation and use of LUS was estimated to cost $38.38 per scan, leading to a cost savings of $37.08 per CXR Portable replaced, and $3.26 per CXR PA/LAT. With 22,273 CXRs ordered to rule out iatrogenic PTX, this represents a total cost reduction of $559,537.10, or an averaged $41.58 of savings per patient.

## 4. Discussion

This single-center retrospective analysis demonstrates that performing CXR to rule out PTX following invasive intrathoracic procedures is resource intensive, with 22,273 CXR ordered for this indication, and economically burdensome with $1.4 million dollars attributed during our study period. As stated above, implementation and substitution with LUS would lead to, a total cost reduction of $559,537.10 and an averaged $41.58 of savings per patient.

With healthcare costs on the rise and financial constraints becoming increasingly prevalent,^[8]^ there is a pressing need to identify opportunities to optimize resource allocation. We have shown that utilizing LUS to rule out PTX following invasive intrathoracic procedures may offer significant cost savings compared to routine CXR. In our sample population, the CXRs ordered represented 8.4% of total CXR completed across the 2 fiscal years. With an estimated over 70 million CXR completed per year in the United States,^[13]^ the magnitude of savings for simple substitution becomes evident. The cost savings of LUS, along with its superior sensitivity and accuracy, further support its broader integration into clinical practice for PTX diagnosis. This shift in the diagnostic paradigm would also eliminate patient exposure to ionizing radiation, which is a commonly cited patient concern.^[14]^

It is also reasonable to assume that the cost of LUS is overestimated, and the implementation of POCUS into clinical workflows would lead to further cost savings. To create a most accurate comparison of CXR costs, we used the same CXR cost-multiplier provided by our institution’s activity-based costing methods. This cost multiplier, in addition to the hardware costs to purchase machines, is likely an overestimate, as the unspecified cost multiplier includes equipment expenses and maintenance. Additionally, a growing body of evidence highlights cost savings in the emergency, trauma, and general medicine departments^[15–19]^ when POCUS is implemented. Therefore, compounded cost savings can be expected outside of our study population as POCUS becomes more widely available and integrated into clinical workflows in other clinical domains.

It should also be noted that our study only analyzed a sample of the population for which LUS could be implemented to rule out PTX. Expanding beyond the subsection of iatrogenic PTX to those with a higher pretest probability of spontaneous PTX or trauma patients may lead to further cost savings. Additionally, most of the cost (64%) per LUS scan is due to the physician fee; however, LUS has been demonstrated to be easy to learn and a superior clinical tool, despite operator.^[6]^ There is potential for substantial cost reduction per thoracic scan by employing alternative operators, such as resident physicians or bedside nursing staff.^[20]^ Additionally, promising research has shown that POCUS assisted by artificial intelligence can further decrease the time spent and enhance the workflow.^[21]^

### 4.1. Limitations

The most notable limitation of this study is its reliance on the case-costing model. Unfortunately, with this delivery of resources at an institutional level, we were unable to acquire granular details about the components that create the cost of CXR delivery. Despite best attempts, this model limits the ability to match the cost components for components with LUS. In addition, there are limitations to capturing our population. We attempted to obtain the most complete sample by focusing on a population that had associated identifiable procedure codes, while also performing an additional search of the CXR indications to minimize missing data. We also introduced uncertainty with the assumption that 2 of the CXR performed during the hospital encounter were for an explicit reason for ruling out a PTX. Although this may limit accuracy, in our clinical experience, it is more likely that we underrepresent the burden of CXRs ordered to rule out PTX in this population. Additionally, limited by our data sample we did not have access to detailed patient information (i.e. patient co-morbidities). We recognize that certain co-morbidities like elevated body-mass-index can make performance of LUS more challenging and based on skill of provider may increase the time component. Lastly, we recognize that the postcardiothoracic surgery population has a more complex pulmonary pathology than other populations, which may introduce uncertainty in LUS findings. However, recent evidence highlights that surgery-related conditions do not impair the sensitivity of LUS.^[22]^

### 4.2. Future directions

As this was a single-center analysis, additional studies documenting the cost of both CXR and LUS in their center or as a multi-center design would be valuable. A prospective design would also allow for the evaluation of CXR activity after the implementation of LUS, to ensure that the assumption of one-to-one replacement is actualized in clinical practice. With previous studies identifying LUS to be easily learned with clinical benefits independent of the operator, there could be further cost-benefits of training bedside nurses in monitored units. Additionally, research focusing on the development and implementation of artificial intelligence associated with LUS may have a significant impact on cost reduction.

## 5. Conclusion

This retrospective economic evaluation comparing LUS and CXR for ruling out PTX in patients undergoing invasive intrathoracic procedures demonstrated cost-saving opportunities associated with LUS employment. Given the superiority of LUS in terms of sensitivity and accuracy for PTX diagnosis, these findings underscore the compelling rationale for its broader integration into clinical practice.

## Author contributions

**Conceptualization:** Carter Winberg, Ross Prager, Chong Sung Kim, Matthew Meyer, Robert Arntfield.

**Data curation:** Carter Winberg, Ross Prager, Chong Sung Kim, Matthew Meyer.

**Formal analysis:** Carter Winberg, Ross Prager, Chong Sung Kim, Matthew Meyer.

**Investigation:** Carter Winberg, Ross Prager, Robert Arntfield.

**Methodology:** Carter Winberg, Ross Prager, Chong Sung Kim, Matthew Meyer, Robert Arntfield.

**Supervision:** Ross Prager, Robert Arntfield.

**Validation:** Carter Winberg, Ross Prager, Chong Sung Kim, Matthew Meyer.

**Writing – original draft:** Carter Winberg, Ross Prager, Robert Arntfield.

**Writing – review & editing:** Carter Winberg, Ross Prager, Chong Sung Kim, Matthew Meyer, Robert Arntfield.

## Supplementary Material



## References

[1] McKnight CL Burns B. Pneumothorax. StatPearls. Treasure Island (FL): StatPearls Publishing; 2023. https://www.ncbi.nlm.nih.gov/books/NBK441885/. 28722915

[2] Al-Hameed FM. Pneumothorax imaging [Medscape web site]. 2022. https://emedicine.medscape.com/article/360796-overview?form=fpf. Accessed May 5, 2024.

[3] Ebrahimi A Yousefifard M Mohammad Kazemi H. Diagnostic accuracy of chest ultrasonography versus chest radiography for identification of pneumothorax: a systematic review and meta-analysis. Tanaffos. 2014;13:29–40. 25852759 PMC4386013

[4] Alrajab S Youssef AM Akkus NI Caldito G. Pleural ultrasonography versus chest radiography for the diagnosis of pneumothorax: review of the literature and meta-analysis. Crit Care. 2013;17:R208. 24060427 10.1186/cc13016 PMC4057340

[5] Aswin K Balamurugan S Govindarajalou R Saya GK Tp E Rajendran G. Comparing sensitivity and specificity of ultrasonography with chest radiography in detecting pneumothorax and hemothorax in chest trauma patients: a cross-sectional diagnostic study. Cureus. 2023;15:e44456. 37791184 10.7759/cureus.44456 PMC10544157

[6] Chan KK Joo DA McRae AD. Chest ultrasonography versus supine chest radiography for diagnosis of pneumothorax in trauma patients in the emergency department. Cochrane Database Syst Rev. 2020;7:CD013031. 32702777 10.1002/14651858.CD013031.pub2 PMC7390330

[7] Lichtenstein DA Mezière G Lascols N. Ultrasound diagnosis of occult pneumothorax. Crit Care Med. 2005;33:1231–8. 15942336 10.1097/01.ccm.0000164542.86954.b4

[8] Canadian Institute for Health Information [CIHI]. National health expenditure trends 2023 snapshot. 2022. https://www.cihi.ca/en/national-health-expenditure-trends-2023-snapshot#:~:text=Total%20health%20expenditure%20expected%20to,19%20pandemic%2C%20particularly%20in%202020. Accessed May 7, 2024.

[9] Rouby JJ Arbelot C Gao Y; APECHO Study Group. Training for lung ultrasound score measurement in critically ill patients. Am J Respir Crit Care Med. 2018;198:398–401. 29557671 10.1164/rccm.201802-0227LE PMC7205011

[10] D’Amato M Rea G Carnevale V. Assessment of thoracic ultrasound in complementary diagnosis and in follow up of community-acquired pneumonia (cap). BMC Med Imaging. 2017;17:52. 28859628 10.1186/s12880-017-0225-5 PMC5579948

[11] Koblan K. How much does an ultrasound machine cost-11 factors to consider [Ultrasound Technology Website]. 2022. https://www.uscultrasound.com/blog/ultrasound-machine-cost/. Accessed June 16, 2024.

[12] Telexy QPathE. Features and costing. 2018. https://www.telexy.com/wp-content/uploads/2018/07/QpathE.pdf. Accessed May 10, 2024.

[13] Rogers M. Routine admission chest x-ray [Core EM web site]. 2017. https://coreem.net/core/routine-admission-cxr-racxr/. Accessed May 12, 2024.

[14] Dauer LT Thornton RH Hay JL Balter R Williamson MJ St Germain J. Fears, feelings, and facts: interactively communicating benefits and risks of medical radiation with patients. AJR Am J Roentgenol. 2011;196:756–61. 21427321 10.2214/AJR.10.5956 PMC3816522

[15] Hirshberg EL Kuttler K Lanspa MJ Brown SM Grissom CK. Ultrasound confirmation of central venous catheter placement could reduce hospital costs. Am J Respir Crit Care Med. 2014;189:A5450.

[16] Testa A Francesconi A Giannuzzi R Berardi S Sbraccia P. Economic analysis of bedside ultrasonography (US) implementation in an internal medicine department. Intern Emerg Med. 2015;10:1015–24. 26450846 10.1007/s11739-015-1320-7

[17] Noritomi DT Zigaib R Ranzani OT Teich V. Evaluation of cost-effectiveness from the funding body’s point of view of ultrasound-guided central venous catheter insertion compared with the conventional technique. Rev Bras Ter Intensiva. 2016;28:62–9. 27096678 10.5935/0103-507X.20160014 PMC4828093

[18] Barchiesi M Bulgheroni M Federici C. Impact of point of care ultrasound on the number of diagnostic examinations in elderly patients admitted to an internal medicine ward. Eur J Intern Med. 2020;79:88–92. 32703675 10.1016/j.ejim.2020.06.026

[19] Lentz B Fong T Rhyne R Risko N. A systematic review of the cost-effectiveness of ultrasound in emergency care settings. Ultrasound J. 2021;13:16. 33687607 10.1186/s13089-021-00216-8 PMC7943664

[20] Corcoran E Hopkins P Fisher R Wong A Rose L. Intensive care nurse-led point of care ultrasound in the assessment and management of the critically ill COVID-19 patient: a single centre case series. Nurs Crit Care. 2023;28:781–8. 36575807 10.1111/nicc.12871 PMC9880746

[21] Mika S Gola W Gil-Mika M Wilk M Misiolłek H. Ultrasonographic applications of novel technologies and artificial intelligence in critically ill patients. J Pers Med. 2024;14:286. 38541028 10.3390/jpm14030286 PMC10971229

[22] Galetin T Merres J Schieren M. Most patient conditions do not a priori debilitate the sensitivity of thoracic ultrasound in thoracic surgery-a prospective comparative study. J Cardiothorac Surg. 2021;16:75. 33849605 10.1186/s13019-021-01454-6 PMC8045207

