# Supplementary material for: Comparative evaluation of lung ultrasound versus chest X-ray for pneumothorax assessment post-invasive intrathoracic procedures: A case-costing evaluation
Source: Medicine (Baltimore). 2025 Apr 25;104(17):e41959. doi: 10.1097/MD.0000000000041959 (PMC12039981; doi:10.1097/MD.0000000000041959)
Supplement: Supplementary file 1 [file medi-104-e41959-s001.docx]

| Appendix Table 1: Cardiothoracic surgeries included in search strategy |
| --- |
| Aortic and Mitral and Tricuspid Valve |
| Aortic and Mitral Valve |
| Aortic and Mitral Valve Redo Sternotomy |
| Aortic Endarterectomy |
| Aortic Root Enlargement |
| Aortic Rupture Repair |
| Aortic Valve |
| Aortic Valve and Ascending Aortic Aneurysm Repair |
| Aortic Valve Bypass |
| Aortic Valve Mini Sternotomy |
| Aortic Valve Mini Thoracotomy |
| Aortic Valve Redo Sternotomy |
| Aortic Valve Transcatheter Apical Insertion Left Anterior Mi |
| Apical Bullectomy VATS |
| Apical Bullectomy VATS Bilateral |
| Ascending Aorta Valve Sparing Root Replacement |
| Ascending Aortic Aneurysm Repair |
| Ascending Aortic Aneurysm Repair Redo Sternotomy |
| Atrial Ablation |
| Atrial Ablation Mini Thorocotamy |
| Atrial Ligation |
| Atrial Septal Defect Repair |
| Atrial Septal Defect Repair with Mini Thoracotomy |
| Axillary Cannulation |
| Bentall Procedure |
| Bentall Procedure Redo Sternotomy |
| Bi-Lobectomy Thoracotomy |
| Bullectomy |
| Bullectomy VATS |
| CABG Double |
| CABG Double Aortic Valve |
| CABG Double Aortic Valve Redo Sternotomy |
| CABG Double Mitral Valve |
| CABG Double Redo Sternotomy |
| CABG Mini Thoracotomy |
| CABG Mini Thoracotomy Robotic |
| CABG Quadruple |
| CABG Quadruple Aortic Valve |
| CABG Quadruple Mitral Valve |
| CABG Quadruple Redo Sternotomy |
| CABG Quintuple |
| CABG Quintuple Mitral Valve |
| CABG Single |
| CABG Single Aortic Valve |
| CABG Single Aortic Valve Redo Sternotomy |
| CABG Single Mitral Valve |
| CABG Single Redo Sternotomy |
| CABG Triple |
| CABG Triple Aortic Valve |
| CABG Triple Aortic Valve Redo Sternotomy |
| CABG Triple Mitral Valve |
| CABG Triple Redo Sternotomy |
| Chest Wall Resection |
| Chest Wall Resection Thoracotomy |
| Claggett Window Creation |
| Decortication and Thoracotomy |
| Decortication Thoracotomy |
| Decortication VATS |
| Diaphragm Hernia Repair Laparoscopy |
| Diaphragm Hernia Repair Laparotomy |
| Diaphragm Hernia Repair Thoracotomy |
| Diaphragm Plication VATS |
| Diverticulectomy Thoracotomy |
| Epicardial Pacemaker Lead Insertion Mini Thoracotomy |
| Epicardial Pacemaker Lead Insertion Subxyphoid Incision |
| Esophageal Fistula Repair |
| Esophageal Gastroscopy |
| Esophageal Myotomy Abdominal |
| Esophageal Myotomy Laparoscopic |
| Esophageal Myotomy Laparoscopic Robotic Assisted |
| Esophageal Myotomy Trans Thoracic |
| Esophagectomy |
| Esophagectomy Laparoscopic Assisted |
| Esophagectomy Laparoscopic Robotic Assisted |
| Esophagectomy Laparoscopy Neck |
| Esophagectomy Laparoscopy Thoracotomy |
| Esophagectomy Laparoscopy VATS |
| Esophagectomy Laparotomy Neck |
| Esophagectomy Laparotomy Thoracotomy |
| Esophagectomy Thoracotomy Laparotomy Neck |
| Esophagectomy Trans Thoracic |
| Esophagectomy VATS |
| Esophagectomy VATS Laparoscopic Assisted |
| Esophagectomy VATS Laparoscopy Neck |
| Exploration Sternotomy |
| Exploration Thoracotomy |
| Exploration VATS |
| Flail Chest Hardware Insertion |
| Flail Chest Hardware Removal |
| Flail Chest Repair Thoracotomy |
| FundoPlication Laparoscopy |
| Gastroplasty Revision |
| Gastroscopy Esophageal Dilatation |
| Gastrostomy |
| Heart Transplant |
| Heart Transplant Redo Sternotomy |
| Heller Myotomy Laparoscopic |
| Hernia Diaphragmatic Repair |
| Hernia Diaphragmatic Repair Laparoscopic |
| Hernia Hiatus Repair |
| Hernia Hiatus Repair Laparoscopic |
| Hernia Hiatus Repair Trans Abdominal |
| Hernia Hiatus Repair Trans Thoracic |
| Hernia Paraesophageal Laparoscopic Robotic Assisted |
| Hernia Paraesophageal Repair |
| Hernia Paraesophageal Repair Laparoscopic |
| Hiatal Hernia Repair Laparoscopy |
| Hiatal Hernia Repair Laparotomy |
| Hiatal Hernia Repair Robotic |
| Hiatal Hernia Repair Thoracotomy |
| Hybrid Total Arch Elephant Trunk Procedure |
| ICD Implant |
| Implantable Cardioverter Defibrillator |
| Internal Thoracic Artery Harvesting |
| Laryngectomy |
| Lobectomy Sleeve Resection Thoracotomy |
| Lobectomy Thoracotomy |
| Lobectomy VATS |
| Lobectomy VATS Robotic Assisted |
| Lung Biopsy or Aspiration Radiology |
| Lung Biopsy Thoracotomy |
| Lung Biopsy VATS |
| Lung Decortication VATS |
| Lung Wedge Excision Thoracotomy |
| Lung Wedge Excision VATS |
| Mediastinal Mass Resection Sternotomy |
| Mediastinal Mass Resection Thoracotomy |
| Mediastinal Mass Resection VATS |
| Mediastinal Tumour Excision |
| Mediastinal Tumour Excision Sternotomy |
| Mediastinal Tumour Excision VATS |
| Mediastinoscopy |
| Mediastinoscopy Neck |
| Mitral and Tricuspid Valve |
| Mitral and Tricuspid Valve Redo Sternotomy |
| Mitral Valve |
| Mitral Valve Redo Mini Thoracotomy |
| Mitral Valve Redo Sternotomy |
| Mitral Valve Repair Mini Thoracotomy |
| Mitral Valve Replacement with Mini Thoracotomy |
| Myectomy Septal |
| Myotomy Laparoscopy |
| Myotomy Laparotomy |
| Myotomy Neck |
| Myxoma Resection |
| Myxoma Resection Redo Sternotomy |
| Myxoma with Mini Thoracotomy |
| Open Cardiac Procedure |
| Pacemaker |
| Pacemaker Generator Replacement |
| Pacemaker Lead Extraction with Fluoroscopy |
| Percutaneous Coronary Intervention |
| Pericardectomy |
| Pericardectomy Radical |
| Pericardial Window Creation |
| Pericardial Window Creation Endoscopic |
| Pericardial Window Creation Thoracotomy |
| Pericardial Window Creation VATS |
| Pericardiocentesis Tamponade |
| Pleural Biopsy Thoracotomy |
| Pleural Biopsy VATS |
| Pleuroscopy |
| Pleuroscopy Pleurodesis Talc Poudrage |
| Pleuroscopy Pleurodesis VATS |
| Pleuroscopy VATS |
| Pleuroscopy VATS & Talc Poudrage |
| Pneumonectomy Thoracotomy |
| Pulmonary Valve |
| Pulmonary Vein Ablation |
| Ross Procedure |
| Segmentectomy Thoracotomy |
| Segmentectomy VATS |
| Sleeve Resection Thoracotomy |
| Sternotomy |
| Sternotomy Redo |
| Sternum Closure with Flap |
| Sternum Debridement |
| Sternum Debridement and Closure |
| Sternum Debridement with Flap Closure |
| Sternum Wire Removal |
| Superior Vena Cava Cannulation |
| Talc Poudrage Thoracotomy |
| Talc Poudrage VATS |
| Thoracic Duct Ligation Thoracotomy |
| Thoracic Sympathectomy |
| Thoracic Window |
| Thoracic Window Thoracotomy |
| Thoracoplasty Thoracotomy |
| Thoracotomy |
| Thoracotomy Mini |
| Thoracotomy Trauma |
| Thoracotomy Ventricular Pacing Lead Placement |
| Thrombectomy |
| Thymectomy Sternotomy |
| Thymectomy Thoracotomy |
| Thymectomy VATS |
| Thymectomy VATS Robotic Assisted |
| Tracheal Resection Neck |
| Tracheal Resection Thoracotomy |
| Tracheal Stenosis Resection |
| Tracheal Tumour Excision |
| Tracheoesophageal Fistula Repair |
| Tracheostomy |
| Tracheostomy Percutaneous |
| Transcatheter Aortic Valve Insertion |
| Tricuspid Valve |
| Tricuspid Valve Redo Sternotomy |
| VATS |
| VATS Robotic Assisted |
| Ventricular Aneurysm Repair |
| Ventricular Aneurysm Repair Redo Sternotomy |
| Wedge Thoracotomy |
| Wedge VATS |
