# Supplementary material for: Comparative evaluation of lung ultrasound versus chest X-ray for pneumothorax assessment post-invasive intrathoracic procedures: A case-costing evaluation
Source: Medicine (Baltimore). 2025 Apr 25;104(17):e41959. doi: 10.1097/MD.0000000000041959 (PMC12039981; doi:10.1097/MD.0000000000041959)
Supplement: Supplementary file 2 [file medi-104-e41959-s002.docx]

| Appendix Table 2: List of words for keyword search of manually entered indications on chest x-ray orders | |
| --- | --- |
| 1 | CT Removal |
| 2 | Off Suction |
| 3 | On Suction |
| 4 | Heimlich |
| 5 | CT insitu |
| 6 | CTx2 |
| 7 | CT reposition |
| 8 | CT daily |
| 9 | Chest tune |
| 10 | CT bilaterally |
| 11 | PTX |
| 12 | PNX |
